# Supplementary material for: Prevalence of Carbendazin Resistance in Field Populations of the Rice False Smut Pathogen Ustilaginoidea virens from Jiangsu, China, Molecular Mechanisms, and Fitness Stability
Source: J Fungi (Basel). 2022 Dec 16;8(12):1311. doi: 10.3390/jof8121311 (PMC9783980; doi:10.3390/jof8121311)
Supplement: Supplementary file 1 [file jof-08-01311-s001.zip › jof-2028275-supplementary.pdf]

Table S1. Deposited DNA sequences of *Uvβ1Tub* and *Uvβ2Tub* of *Ustilaginoidea virens* isolates in GenBank.

Species	Gene ID	Isolate	Accession Numbers
Ustilaginoidea virens	UV8b_05680	HWD	OP379799
	UV8b_05680	JS60	OP379800
	UV8b_05680	JY7b	OP379801
	UV8b_05680	JY11a	OP379802
	UV8b_05680	JY30b	OP379803
	UV8b_05680	GY13c	OP379804
	UV8b_05680	GY14b	OP379805
	UV8b_05680	GY20a	OP379806
	UV8b_05680	GY47c	OP379807
	UV8b_05680	GY64a	OP379808
	UV8b_05680	GY65b	OP379809
	UV8b_05680	JY4a	OP379810
	UV8b_05680	JY18b	OP379811
	UV8b_05680	JY20a	OP379812
	UV8b_05680	JY22a	OP379813
	UV8b_05680	JY24b	OP379814
	UV8b_05680	JY33d	OP379815
	UV8b_05680	XH3a	OP379816
	UV8b_05680	XH33c	OP379817
	UV8b_05680	XH36b	OP379818
	UV8b_05680	XH39c	OP379819
	UV8b_05680	XH46a	OP379820
	UV8b_05680	XH57a	OP379821
	UV8b_05680	HA26	OP379822
	UV8b_05680	XH7b	OP379823
	UV8b_05680	ZJ24	OP379824
	UV8b_05680	GL11	OP379825
	UV8b_05680	GL12b	OP379826
	UV8b_05680	GL23	OP379827
	UV8b_05680	HA17	OP379828
	UV8b_05680	XH43b	OP379829
	UV8b_05680	JR11	OP379830
	UV8b_05680	JR12	OP379831
	UV8b_05680	YD8	OP425142
	UV8b_05680	ZJ7	OP425143
	UV8b_05680	YZ11	OP425144
	UV8b_05680	XH5a	OP425145

UV8b_05383	GL11	OP425813
UV8b_05383	GL12b	OP425814
UV8b_05383	GL23	OP425815
UV8b_05383	HA26	OP425816
UV8b_05383	JR11	OP425817
UV8b_05383	JR12	OP425818
UV8b_05383	XH5a	OP425819
UV8b_05383	XH7b	OP425820
UV8b_05383	XH43b	OP425821
UV8b_05383	YD8	OP425822
UV8b_05383	YZ11	OP425823
UV8b_05383	ZJ7	OP425824
UV8b_05383	ZJ24	OP425825
UV8b_05383	JY7b	OP425826
UV8b_05383	JY11a	OP425827
UV8b_05383	JY30b	OP425828
UV8b_05383	GY13c	OP425829
UV8b_05383	GY14b	OP425830
UV8b_05383	GY20a	OP425831
UV8b_05383	GY47c	OP425832
UV8b_05383	GY64a	OP425833
UV8b_05383	GY65b	OP425834
UV8b_05383	JY4a	OP425835
UV8b_05383	JY18b	OP425836
UV8b_05383	JY20a	OP425837
UV8b_05383	JY22a	OP425838
UV8b_05383	JY24b	OP425839
UV8b_05383	JY33d	OP425840
UV8b_05383	XH3a	OP425841
UV8b_05383	XH33c	OP425842
UV8b_05383	XH36b	OP425843
UV8b_05383	XH39c	OP425844
UV8b_05383	XH46a	OP425845
UV8b_05383	XH57a	OP425846
UV8b_05383	HWD	OP425847
UV8b_05383	JS60	OP425848
UV8b_05383	HA17	OP425849
